# Community and societal influences on loneliness and social isolation among young adults: a systematic review of observational studies

**DOI:** 10.1093/epirev/mxag007

**Published:** 2026-03-16

**Authors:** Drew Eleanor Meehan, Anne Grunseit, Neta HaGani, Dafna Merom

**Affiliations:** School of Health Sciences, Western Sydney University, Campbelltown, New South Wales, Australia; Department of Public Health, School of Psychology and Public Health, La Trobe University, Melbourne, Victoria, Australia; School of Public Health, Faculty of Health, University of Technology Sydney, Sydney, New South Wales, Australia; Sydney School of Public Health, Faculty of Health, University of Sydney, Camperdown, New South Wales, Australia; Prevention Research Collaboration, School of Public Health, University of Sydney, Camperdown, New South Wales, Australia; School of Health Sciences, Western Sydney University, Campbelltown, New South Wales, Australia

**Keywords:** loneliness, social isolation, young adults, community determinants, social-ecological model

## Abstract

Loneliness and social isolation are emerging public health concerns among young adults, yet the role of community and societal factors remains poorly understood. Existing research has predominantly focused on individual or interpersonal determinants. This systematic review synthesized observational evidence on community- and societal-level factors associated with loneliness and social isolation among young adults aged 18-30 years. Five databases were searched for observational studies examining place-based community or societal exposures and loneliness or social isolation outcomes. Thirteen eligible studies were identified, and a structured narrative synthesis was undertaken. The exposure variables explored were diverse. Neighborhood characteristics (including trust, safety, and atmosphere), community participation, cultural and societal norms, and structural conditions demonstrated consistent associations with loneliness. Greater neighborhood cohesion, safety, and belonging were protective, whereas neighborhood disorder, minority status, perceived discrimination, and individualistic cultural orientations were associated with greater loneliness. Evidence for social isolation was sparse and methodologically heterogeneous, though area-level disadvantage and remoteness showed emerging relevance. Most studies had a cross-sectional design, and the ways loneliness and social isolation were measured across studies were heterogenous. Community and societal determinants meaningfully shape young adults’ experiences of loneliness, but evidence for social isolation remains limited. Findings highlight the need for longitudinal research, improved consistency in the use of measurement tools, and further examination of moderators between individual factors and community influences, which will all contribute to the development of multilevel public health strategies addressing structural and neighborhood conditions.

## Introduction

Loneliness and social isolation are important determinants of mental and physical well-being across the lifespan. In young adults, also termed emerging adults and here defined as individuals aged 18 to 30 years, these experiences are strongly associated with adverse mental health outcomes. Systematic reviews have demonstrated strong relationships among loneliness, social isolation, and suicidality, as well as increased risk of depression and anxiety.^1^^,^^2^ In later life, chronic loneliness and social isolation have been linked to elevated risk of dementia and cardiovascular disease.^3-5^ Given the well-documented health implications of loneliness and social isolation, there is a need to develop comprehensive public health approaches to prevention.^6^

Conceptually, loneliness and social isolation represent distinct but related experiences.^7^ Loneliness, also termed as subjective or perceived isolation, refers to “the subjective unpleasant or distressing feeling of a lack of connection to other people, along with a desire for more, or more satisfying, social relationships.”^8^ Conversely, social isolation, or objective isolation, denotes “having objectively few social relationships, social roles and group memberships, and infrequent social interaction.”^8^ Whether experienced concurrently, or independently, loneliness and social isolation may contribute to negative mental health outcomes in young adults.^9^

Contrary to earlier assumptions that loneliness and social isolation predominantly affect older adults due to life transitions such as retirement and bereavement, recent global policy frameworks recognize loneliness and social isolation as public health concerns across the life course, with young people identified as a priority population for prevention efforts.^10^ Despite this, research and intervention efforts have typically focused disproportionately on older populations^11^ with comparatively limited attention to younger adults. This gap is particularly concerning given that young adulthood is a formative developmental period during which social trajectories and health-related behaviors are established.

The social ecological model provides a valuable framework for understanding complex public health issues by examining multiple levels of influence. We use the conceptual framework of Dahlberg and Krug,^12^ which delineates 4 distinct levels of interaction: individual (personal biological and psychological factors), interpersonal (close relationships that influence behavior), community (settings and shared environments that enable social interaction), and the societal (broader cultural and policy factors). This framework guides our investigation of loneliness and social isolation as multilevel phenomena.

Although existing systematic reviews investigating loneliness and social isolation in young adults focused on individual and interpersonal factors, such as their parental relationships and personal identity,^13^^,^^14^ our review addresses the less-researched community and societal determinants (eg, neighborhood characteristics, social cohesion, cultural norms). This approach aligns with emerging research directions in the older adult population^15^ and addresses a significant gap in understanding the multilevel influences on young adult social connection.

We aim to identify and synthesize the range of community and societal determinants of loneliness and social isolation among young adults. We seek to establish a more robust foundation for developing targeted, multilevel interventions to address these significant public health challenges among young adults.

## Methods

### Study design and reporting

This systematic review follows the JBI methodology for systematic reviews, and items are reported against the Preferred Reporting Items for Systematic reviews and Meta-Analyses (PRISMA) 2020 Statement.^16^ The PRISMA reporting items are available in Table S1. This systematic review was registered prospectively in the Prospective Register of Systematic Reviews (PROSPERO registration CRD42024509049).

### Search strategy

The search strategy was designed in collaboration with a health science research librarian using the population, exposure, comparison, outcomes, and study design (PECOS) framework. Given the exploratory aim of identifying community and societal influences, exposure-related keywords (eg, “neighborhood,” “community cohesion,” “policy,” “social media”) were not applied. Instead, exposures were identified inductively from included studies. Full search strategies for each database are available in Tables S2-S6.

The searches were undertaken in the Embase (OvidSP), MEDLINE (Ovid), APA PsycInfo (EBSCOhost), CINAHL Plus (EBSCOhost), and Scopus databases. Searches were conducted between February 2024 and April 2024 and were re-run in October 2025 to ensure contemporary studies were appropriately included, given this is a rapidly evolving field. Google Scholar was searched for unique entries using keywords, and further hand-searching was completed using forward and backward citation tracking of included articles.

Search results were uploaded to Covidence^17^ and duplicates were removed at this stage. Covidence was used for title and abstract screening by 1 reviewer. Full texts were screened for relevance by 2 reviewers, and any conflicts were resolved by a third reviewer if they could not be resolved by discussion.

### Eligibility criteria

Inclusion and exclusion criteria were determined using the PECOS framework (Table 1). Studies were included if they used an observational data collection method. Included studies were required to sample predominantly young adults, defined as individuals 18-30 years old. Where age ranges were reported without a mean age, studies were included if the reported age band substantially overlapped with this developmental period. Where mean age was available, inclusion was guided by a mean between 22 and 26 years (midpoint of the target age range ± 2 years). This approach was necessary because several large population-based surveys report broader age categories that encompass emerging adulthood without providing narrower age stratification.

**Table 1:** PECOS parameters for inclusion and exclusion criteria.

**PECOS**	**Inclusion criteria**	**Exclusion criteria**
Population	Young adults aged between 18 and 30 years, or where an age range is not given, the mean age should be between 22 and 26 years	Young adults who are institutionalized or incarcerated
Exposure	Community or societal level factors or measures (eg, social cohesion, neighborhood safety)	–
Comparison	–	–
Outcome	Measures of loneliness and/or social isolation, regardless of the tool used	–
Study design	• Observational • Cross-sectional • Case–control • Prospective cohort • Retrospective/historical cohort	• Reviews, books, opinion pieces, editorials, protocols • Observational studies using aggregated data (eg, ecological studies) • Qualitative studies • Intervention studies. • Published in a language other than English

Studies eligible for inclusion operationalized loneliness and/or social isolation as outcomes. Furthermore, eligible studies examined community- or societal-level exposures, per the Dahlberg and Krug^12^ social ecological model, defined as factors reflecting characteristics of local settings, institutions, social environments, or broader structural contexts (eg, neighborhood quality, social cohesion, remoteness, cultural norms, participation in community-based institutions). Classification of exposures was based on how variables were operationalized within the original studies, and their corresponding survey instruments. Exposures were classified at the community level when they represented engagement with or characteristics of shared social environments, rather than purely dyadic interpersonal relationships (ie, relationship quality with a specific partner, parent, or friend). Studies that only included measures of individual (eg, heath status, education, income) and/or interpersonal level (eg, household structure, parental relationships) exposures were excluded. All included studies had to be peer-reviewed original research. Reviews, books, opinion pieces, editorials, protocols, and gray literature were not included.

### Data extraction

Data extraction and critical appraisal were completed concurrently using Excel.^18^ Detailed data extraction criteria were developed to maintain consistency and were tested on a subsample (15%; *n* = 2) of included articles to assess clarity, completeness, and applicability of the extraction framework. The pilot articles were selected based on alphabetical order of the first author’s surname. This pilot phase enabled refinement of variable definitions and extraction categories prior to full data extraction across all included studies. The process resulted in the inclusion of 1 additional extraction criterion (conflicts of interest) but found the extraction tool to be a good fit across all domains. Data extraction included author names; publication year; journal; conflicts of interest; funding source; aim of study; study design; recruitment strategy; data collection method; age range or mean age of participants (both where available); country of study (including specific locations where applicable); sample size; percentage of participants who were female; outcome and measurement tool; exposures and measurement tools; confounders; statistical methods; main findings; and recommendations for future research. Effect sizes were recorded as provided in the articles, with adjusted results being preferred to account for confounding, where possible.^19^ Additionally, a record of any comments made by the extractor was made. To critically evaluate the included articles, the JBI critical appraisal checklist for analytical cross-sectional studies and the JBI critical appraisal checklist for cohort studies were used.^20^

### Data synthesis

A narrative synthesis of the studies was conducted in several structured stages. First, extracted data were tabulated to enable comparison of study characteristics, measurement approaches, and key findings. Second, studies were grouped inductively according to the ecological level of the determinant examined (eg, community infrastructure, neighborhood social norms, policy, structural factors). Within each group, patterns of association were identified by comparing the direction, magnitude, and consistency of findings, with attention to whether estimates were adjusted for relevant confounders.

## Results

### Article characteristics

**Figure 1:**
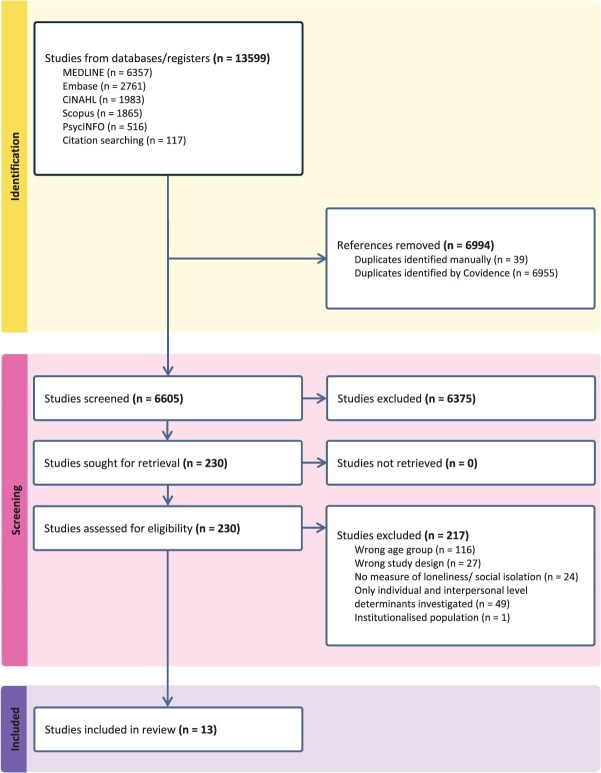
Preferred Reporting Items for Systematic reviews and Meta-Analyses (PRISMA) flow diagram including study identification, screening processes, and included articles.

As presented in the PRISMA diagram in Figure 1, there were 13 482 entries returned from the detailed search strategy, along with 117 additional articles found through citation searches. From the total, 6994 duplicates were removed, leaving 6605 articles for title and abstract screening. Of these, 230 articles ultimately underwent full-text screening, which was completed independently by 2 researchers. Reasons for article exclusion were recorded and are displayed in Figure 1. Thirteen articles met the eligibility criteria. All were published between 2016 and 2025^21-32^; there were 12 cross-sectional studies and 1 cohort study.^32^

Of the 13 included articles, 4 reported on studies conducted solely in the United States.^28-31^ Three were based in the United Kingdom,^24^^,^^26^ including 1 that focused specifically on England and Wales.^24^ Two studies were based in Israel,^23^^,^^27^ 2 in Australia,^22^^,^^32^ and 1 each in Finland^21^ and Malaysia.^25^

Outcomes of the different studies were reported as follows: 1 study exclusively assessed social isolation^30^; 1 study assessed both loneliness and social isolation at 2 time points^31^; and another cohort study assessed loneliness and social isolation concurrently.^32^ The remaining 10 studies exclusively assessed loneliness.^21-29^^,^^33^

To measure loneliness, 5 of 12 articles reported on studies that used a variation of the University of California, Los Angeles (UCLA) Loneliness Scale (UCLA-LS).^24-26^^,^^29^^,^^30^ Another 5 of 12 articles reported on studies that used a single-item direct question to estimate loneliness,^21^^,^^23^^,^^27^^,^^31^^,^^33^ another study used the De Jong-Gierveld Loneliness Scale (DJG-LS),^22^ and the remaining 1 used other methods.^32^ To measure social isolation, 1 study used the Berkman-Syme Social Network Index,^30^ 1 used an unvalidated single-item question to assess social isolation,^31^ and another study used a previously validated, but not widely used, scale.^32^ Further descriptions of the articles can be found in Tables 2 and 3.

**Table 2:** Summary of included articles.

**Study author and design**	**Aim**	**Study sample size; age range; % female participants**	**Country**	**Data collection method and year**	**Loneliness tool**	**Social isolation tool**	**Community/ societal level determinant (measurement tool)**	**Included covariates**
Achdut and Refaeli^23^ Cross-sectional	To explore inequalities in loneliness among 3 ethnocultural groups in Israel: native Jewish people, immigrants from the former Soviet Union, and Arab people	4253; 20-34; not stated; 49.8	Israel	Self-report questionnaires administered by interviewers (Israeli Social Survey) (2016-2017)	Single item: Do you ever feel lonely?	N/A	• Ethnocultural group (immigration status) • Perceived discrimination (single-item question) • Neighborhood social capital (4-item scale)	Age, gender, marital status, parenthood, education level and “not in education, employment or training” status
Carbonaro^31^ Cross-sectional	To examine 2 mechanisms by which police contact contributes to deleterious health outcomes: system avoidance and social isolation	12 016; 18-27; 22; 55	United States	School-administered survey (National Longitudinal Study of Adolescent to Adult Health) (1994-2009)	Single item: How often do you feel isolated from others?	Single item: How many times during the past week did you just hang out or talking with your friends?	• Police stops (single-item question)	Gender, ethnicity, age, parental education, educational attainment, health insurance, immigration status
Chen et al.^28^ Cohort	To analyze longitudinal data from 3 large cohorts of young, middle-aged, and older adults, with repeated measurements of religious-service attendance and multiple health and well-being outcomes	9862; not stated; 23.0 at baseline; 59.4-67.9	United States	Self-report survey (Growing Up Today Study) (2007-2016)	Single item: I felt lonely	N/A	• Religious service attendance (single-item question)	Age, gender, race/ethnicity, marital status, geographic region, employment status, nightshift work schedule, socioeconomic status, health-insurance status, childhood maternal attachment and childhood-abuse victimization
Goh et al.^25^ Cross-sectional	To determine whether cultural orientation and gender would moderate the positive association between loneliness and socio-sexuality	319; 18-30; not stated; 58	Malaysia	Online survey (2020)	6-Item UCLA Loneliness Scale	N/A	• Cultural orientation (individualism and collectivism scale)	Age, sex, ethnicity, sexual orientation, partnered sexual experience, relationship status, socioeconomic status, religiosity
Goodfellow et al.^33^ Cross-sectional	To examine the extent to which loneliness is related to personal well-being in older young people specifically, and to determine how this relationship differs depending on social-ecological factors	4253; 16-24; not stated; 58	United Kingdom	Self-report using online or paper survey (Community Life Survey) (2017-2018)	Single item: How often do you feel lonely?	N/A	• Neighborhood belonging (single-item question) • Neighborhood trust (single-item question)	Moderating personal well-being
Gregory et al.^22^ Cross-sectional	To investigate the relationship between social media use and loneliness and psychological well-being of youth in rural NSW	47; 16-24; not stated; 68.1	Australia (NSW regional areas only)	Online survey (2021-2022)	6-Item De-Jong Gierveld Loneliness Scale	N/A	• Rurality (Modified Monash Model)	Gender, age, household composition, employment status, educational level, general state of health
Marquez et al.^26^ Cross-sectional	To determine social-ecological predictors of loneliness, and the extent to which geographic region may account for differences in loneliness among young people in the United Kingdom	6503; 16-24 ; 19; 55.5	United Kingdom	Annual household survey (UK Household Longitudinal Study- Understanding Society) (2017-2019)	3-Item UCLA Loneliness Scale	N/A	• Perceived neighborhood quality (9-item scale) • Neighborhood belonging (single-item question) • Being similar to others in the neighborhood (single-item question) • Talking regularly with neighbors (single-item question) • Having local friends (single-item question) • Community type (Census 2001 Output Area Classification)	Age, gender, sexual orientation, ethnicity, religiosity, country of residence, remoteness, subjective financial situation
Matthews et al.^24^ Cross-sectional	To investigate how aspects of neighborhoods relate to young adults’ feelings of social disconnection	2130; 18-19; not stated; 53	England and Wales	Home interview (Environmental Risk (E-Risk) Longitudinal Twin Study) (2012-2013)	4-Item UCLA Loneliness Scale	N/A	• Population density (number of people in a 0.5-mile radius from the home address) • Neighborhood street safety (physical inspection using Google Street View) • Neighborhood disorder (physical inspection using Google Street View) • Urbanicity (Office for National Statistics’ 2011 Rural-Urban Classification) • Social cohesion (Sampson, Raudenbush^34^ social cohesion scale)	Depression, neuroticism, living with a twin, sex, socioeconomic status
Meehan et al.^32^ Cohort	To identify community-level determinants of loneliness and social isolation and compare their effects among younger and older adults within a population cohort in Australia	4623; 18-30; not stated; ~ 50%	Australia	Home interview and online survey (2006-2018)	3-item Sydney University Loneliness Scale	4-item Sydney University Social Isolation Scale	• Civic engagement (4-item scale) • Community engagement (5-item scale) • Altruism (2-item scale) • Cultural practices (2-item scale) • Neighborhood safety (4-item scale) • Neighborhood social cohesion (Sampson, Raudenbush^34^ social cohesion scale) • Neighborhood atmosphere (3-item scale) • Remoteness (Australian Statistical Geography Standard, 2011 Remoteness Areas) • Socioeconomic Index for Area quintile^36^	Age, gender, ethnicity, marital status, obtained level of education, self-assessed health, number of people in dwelling, working status, and gross annual household income
Nyqvist et al.^21^ Cross-sectional	To examine the association between social capital and experienced loneliness in different age groups in a Finnish setting	774; 15-29; Not stated; 60.5	Finland (western)	Postal survey (2011)	Single item: Do you feel lonely?	N/A	• Organizational activities (single-item question) • Neighborhood trust (single-item question) • Neighborhood belonging (single-item question)	Age, gender, marital status, basic education and language
Refaeli and Achdut^27^ Cross-sectional	To examine the associations among perceived poverty, perceived income adequacy, social capital (including online social capital), and neighborhood capital and loneliness, and to examine whether social or neighborhood capital are protective factors that moderate the association between perceived poverty, perceived income adequacy, and loneliness	1508; 20-29; not stated; 49	Israel	Self-report questionnaires administered by interviewers (Israeli Social Survey) (2017)	Single item: Do you ever feel lonely?	N/A	• Neighborhood trust (single-item question) • Neighborhood safety (single-item question) • Noise (single-item question) • Air pollution (single-item question)	Age, gender, ethnicity, immigration, marital status, parenthood, education level, employment status, and physical health problems
Rovito et al.^30^ Cross-sectional	To describe the findings pertaining to social isolation as an indicator of the structural dimension of social connectedness	495; 18-25; 21.78 ± 1.86; 0	United States	Online survey (2019)	N/A	Berkman Syme Social Network Index	• Poverty in neighborhood (government data) • Life expectancy in area (2018 County Health Rankings and Roadmaps program) • Masculinity; physiological energy (4-item adapted scale from Perceived Masculinity Scale-47) • Masculinity; idealized gender (2-item adapted scale from Perceived Masculinity Scale-47)	Age, sexuality, ethnicity, education, religiosity, marital status, adverse childhood experiences
Rovito et al.^29^ Cross-sectional	To examine select social-ecological correlates of loneliness in young adult men in the United States	495; 18-25; 21.78 ± 1.86; 0	United States	Online survey (2019)	3-Item UCLA Loneliness Scale	N/A	• Poverty in neighborhood (government data) • Life expectancy in area (2018 County Health Rankings and Roadmaps program) • Masculinity; physiological energy (4-item adapted scale from Perceived Masculinity Scale-47) • Masculinity; idealized gender (2-item adapted scale from Perceived Masculinity Scale-47)	Age, sexuality, ethnicity, education, religiosity, marital status, adverse childhood experiences

Abbreviations: N/A, not applicable; NSW, New South Wales; UCLA, University of California, Los Angeles.

**Table 3:** Direction and significance of factors associated with loneliness.

**Domain**	**Factor**	**Measurement tool**	**Association with loneliness (source)**	**Association with social isolation (source)**
Neighborhood Characteristics	Neighborhood belonging	Single-item question	Negative association^*^^21^^,^^26^	N/A
	Neighborhood trust	Single-item question	Negative association^*^^21^^,^^27^	N/A
	Neighborhood safety	Single-item question	Negative association^*^^27^^,^^32^	Negative association^*^^32^
	Bothered by noise	Single-item question	Positive association*^27^	N/A
	Bothered by air pollution	Single-item question	Positive association*^27^	N/A
	Being similar to others in the neighborhood	Single-item question	Negative association*^26^	N/A
	Perceived neighborhood quality	9-item scale (not validated)	Negative association*^26^	N/A
	Community type (multicultural community)	Census 2001 Output Area Classification	Positive association*^26^	N/A
	Population density	Number of people in a 0.5-mile radius from the home address	Negative association^24^	N/A
	Neighborhood street safety	Physical inspection using Google Street View	Negative association^24^	N/A
	Neighborhood disorder	Physical inspection using Google Street View	Positive association^24^	N/A
	Social cohesion (collective efficacy)	Sampson, Raudenbush^34^ social cohesion scale	Negative association*^24^	Negative association*^32^
	Neighborhood social capital (increased social capital)	4-item scale	Negative association*^23^	N/A
Community Participation	Cultural practices, including religious service attendance and organizational activities	Single-item question, 2-item scale	Negative association*^21^^,^^28^^,^^32^	N/A
	Community engagement including having local friends, and talking with neighbors	Single-item question, 5-item scale	Negative association*,^26^^,^^32^ Positive association^26^	Negative association*^32^
Cultural and societal norms	Ethnocultural group (minority population)	Immigration status	Positive association*^23^	N/A
	Cultural orientation (individualism)	Individualism and collectivism scale	Positive association*^25^	N/A
	Masculinity; physiological energy	4-item adapted scale from Perceived Masculinity Scale-47	Positive association*^29^	Negative association^30^
	Masculinity; idealized gender	2-item adapted scale from Perceived Masculinity Scale-47)	Positive association^29^	Negative association^30^
	Perceived discrimination	Single-item question	Positive association*^23^	N/A
Structural and systemic factors	Poverty in neighborhood	Government data	Positive association^29^	Positive association*^30^
	Life expectancy in area	2018 County Health Rankings and Roadmaps program	Negative association^29^	Positive association^30^
	Police stops	Single-item question	Positive association*^31^	Positive association^31^
Remoteness	Urbanicity	Office for National Statistics’ 2011 Rural–Urban Classification	Positive association^24^	N/A
	Rurality	Modified Monash Model, Australian Statistical Geography Standard, 2011 Remoteness Areas	Negative association^22^ Positive association*^32^	Positive association*^32^

^*^Study reported the results as significant at *P* < 0.05.

Abbreviations: N/A, not applicable.

Community and societal factors were measured in the included articles, with 5 key domains of exposure measures. Neighborhood characteristics (evaluated in 5 studies)^24^^,^^26^^,^^27^^,^^32^^,^^33^ included factors like neighborhood safety, trust, and belonging, and similarity to others in the neighborhood. Four studies looked at community participation and resources^21^^,^^24^^,^^28^^,^^32^ and included cultural practices like religious attendance and organizational activities, and community engagement such as having local friends and talking with neighbors. Remoteness, assessed in 3 studies,^22^^,^^24^^,^^32^ included comparisons between urban and remote areas. Cultural and societal norms (*n* = 5 studies^23^^,^^25^^,^^26^^,^^29^^,^^30^) included factors like minority groups, masculinity, and individualism. Finally, structural and systemic factors like poverty and police stops were explored by 3 studies.^29-31^ Although some survey questionnaires included questions about religious service attendance or having local friends, which were reported at the individual level, they were conceptualized by the researchers of the included study as indicators of engagement with community institutions or neighborhood social environments and therefore were classified as community-level exposures, consistent with the social ecological framework applied in this review. All included articles (*n* = 13) also controlled for factors at the individual and interpersonal levels, commonly incorporating age, gender, education, and income.^21-33^^,^^35^

### Critical appraisal

The critical appraisal assessments are presented in Table 4. The JBI recommends no further aggregating of the critical appraisal scores be undertaken. There were 8 studies, including the 1 cohort study,^32^ that met all the criteria, indicating high quality.^21^^,^^22^^,^^24^^,^^26^^,^^27^^,^^29^^,^^30^ One study did not meet the criteria in more than half the domains, thus, the results of that study^28^ should be interpreted with caution.

**Table 4:** Critical appraisal of included studies using JBI tools.

**Included Study**	**Criteria**										
	**Were the criteria for inclusion in the sample clearly defined?**	**Were the study subjects and the setting described in detail?**	**Was the exposure measured in a valid and reliable way?**	**Were objective, standard criteria used for measurement of the condition?**	**Were confounding factors identified?**	**Were strategies to deal with confounding factors stated?**	**Were the outcomes measured in a valid and reliable way?**	**Was appropriate statistical analysis used?**			
Cross-sectional studies											
Achdut and Refaeli^23^	Y	Y	Y	Y	N	N	Y	Y			
Carbonaro^31^	Y	N	Y	Y	Y	Y	N	Y			
Chen et al.^28^	N	N	U	U	Y	Y	U	Y			
Goh et al.^25^	Y	N	Y	Y	Y	Y	Y	Y			
Goodfellow et al.^33^	Y	N	Y	Y	Y	Y	Y	Y			
Gregory et al.^22^	Y	Y	Y	Y	Y	Y	Y	Y			
Marquez et al.^26^	Y	Y	Y	Y	Y	Y	Y	Y			
Matthews et al.^24^	Y	Y	Y	Y	Y	Y	Y	Y			
Nyqvist et al.^21^	Y	Y	Y	Y	Y	Y	Y	Y			
Refaeli and Achdut^27^	Y	Y	Y	Y	Y	Y	Y	Y			
Rovito et al.^30^	Y	Y	Y	Y	Y	Y	Y	Y			
Rovito et al.^29^	Y	Y	Y	Y	Y	Y	Y	Y			
	**Were the two groups similar and recruited from the same population?**	**Were the exposures measured similarly to assign people to both exposed and unexposed groups?**	**Was the exposure measured in a valid and reliable way?**	**Were confounding factors identified?**	**Were strategies to deal with confounding factors stated?**	**Were the groups/participants free of the outcome at the start of the study (or at the moment of exposure)?**	**Were the outcomes measured in a valid and reliable way?**	**Was the follow up time reported and sufficient to be long enough for outcomes to occur?**	**Was follow up complete, and if not, were the reasons to loss to follow up described and explored?**	**Were strategies to address incomplete follow up utilized?**	**Was appropriate statistical analysis used?**
Cohort study											
Meehan et al.^32^	N/A	N/A	Y	Y	Y	Y	Y	Y	Y	Y	Y

Abbreviations: N, not present; U, unclear; Y, present.

### Narrative synthesis

We have organized the community and societal determinants by 5 key themes from the included studies. These are further summarized in Table 3.

#### Neighborhood characteristics

Neighborhood factors emerged as 1 of the most consistently studied determinants of loneliness in young adults, with 13 distinct factors examined across studies (Table 3). Among these, neighborhood trust, safety, and belonging had demonstrated evidence of association, with each factor having significant inverse associations with loneliness in multiple studies.^21^^,^^26^^,^^27^^,^^32^

Refaeli and Achdut^27^ found clear inverse relationships among neighborhood trust, neighborhood safety, and loneliness and a positive relationship between atmosphere factors (including noise and pollution) and loneliness. These findings were corroborated by Marquez et al.,^26^ who reported that neighborhood belonging, perceived similarity to others in the neighborhood, and neighborhood quality were all inversely associated with loneliness. Marquez et al.^26^ also found that living in a multicultural community was significantly associated with increased loneliness. This finding is consistent with their observation that perceived similarity to others in the neighborhood was inversely associated with loneliness, suggesting that perceived social homogeneity may play an important role in young adults’ feelings of connection within their communities.

Matthews et al.^24^ presented somewhat contradictory findings regarding population density, showing a negative but nonsignificant association with loneliness. In the same study, social cohesion emerged as a significant protective factor against loneliness, independent of population density. This suggests the quality of social connections within neighborhoods may be more influential than the sheer number of people residing in an area.

#### Community participation and resources

Community participation emerged as another domain with consistent evidence, with measured factors showing significant associations with loneliness (Table 3). For example, cultural practices, which included religious service attendance, and participation in organizational activities demonstrated inverse relationships with loneliness,^21^^,^^28^^,^^33^ suggesting that structured community participation serves as a protective factor against loneliness in young adults. Goodfellow et al.^33^ similarly found that community involvement can mediate the relationship between loneliness and personal wellbeing. Interestingly, Marquez et al.^26^ found that having local friends had a positive but nonsignificant association with loneliness.

#### Cultural and societal norms

Four studies examined various aspects of cultural influence on loneliness, with 4 of 5 factors demonstrating significant associations with loneliness (Table 3). For example, building on the findings of Marquez et al.^26^ regarding multicultural communities, Achdut and Refaeli^23^ found that individuals of ethnic minorities are more susceptible to loneliness than those in the population majority. In a Malaysian study, Goh et al.^25^ found that people who displayed strong cultural individualism were more susceptible to loneliness. Rovito et al.^29^ found a significant relationship between idealized masculinity and loneliness in a male sample, suggesting that restrictive societal expectations regarding gender expression may contribute to feelings of social disconnection. In an earlier study, Rovito et al.^30^ found that this idealized masculinity may reduce social isolation, highlighting differential effects for each outcome.

#### Structural and systemic factors

Structural and systemic factors demonstrated mixed patterns of association with loneliness and social isolation (Table 3). For social isolation, 1 study found that greater area poverty rates were associated with increased social isolation, pointing out the role of socioeconomic inequalities in shaping social connection opportunities.^30^ Carbonaro^31^ investigated the effects of police stops on loneliness and social isolation, and found that more frequent stops significantly increased loneliness, potentially through stigmatizing processes. However, no significant relationship was found between police stops and social isolation,^31^ highlighting the distinct nature of these 2 outcomes.

#### Remoteness

Remoteness was the least studied domain, with only 3 studies examining geographic influences on loneliness (Table 3). In an Australian context, Gregory et al.^22^ found no significant association between loneliness and rurality; however, Meehan et al.,^32^ using a longitudinal design, found a weak positive association between rurality and loneliness and a similar association with social isolation. In the British context, Matthews et al.^24^ reported urbanicity had no significant effect on loneliness in young adults, challenging common assumptions about urban-rural differences in social connection for young adults.

## Discussion

To our knowledge, this review is the first systematic examination of community and societal determinants of loneliness and social isolation specifically in young adults (aged 18-30 years). Our analysis identified 5 key domains of community and societal factors investigated in relation to the loneliness and social isolation of interest, namely neighborhood characteristics, community participation and resources, cultural and societal norms, structural and systemic factors, and remoteness. Higher neighborhood quality, including increased neighborhood atmosphere, greater neighborhood safety, and stronger social cohesion, were found to be protective against loneliness. Conversely, neighborhood disorder and being part of a minority group were associated with higher levels of loneliness. Although fewer studies investigated social isolation as an outcome, higher area poverty rates were significantly associated with increased social isolation, whereas stronger masculine identity showed a protective relationship in a cohort of men aged 18-25 years. Notably, nearly three-quarters of the included studies were published since 2020, underscoring that this field remains in its infancy despite growing recognition of the public health importance of these issues among young adults. Next, we examine the implications of these findings for measurement approaches, discuss potential mechanisms underlying observed associations, and identify critical directions for future research and intervention development.

The scope of this review was intentionally restricted to place-based community and societal determinants; therefore, we did not include studies focused primarily on digital or online social environments, nor studies examining the impacts of the COVID-19 pandemic. These exclusions were methodological rather than conceptual. First, digital determinants, including social media use, online communication patterns, and technology-mediated social connection, have been comprehensively synthesized elsewhere, with recent systematic reviews demonstrating complex, bidirectional associations between digital engagement and mental health outcomes, including loneliness in young adults.^37–39^ Second, a rapidly expanding evidence base has examined the effects of COVID-19 restrictions on loneliness and social isolation; systematic reviews consistently show elevated prevalence across age groups and document pandemic-specific mechanisms.^40^^,^^41^ Including these published reports would have broadened the review beyond its core theoretical aim of understanding place-based community and societal determinants in nonpandemic contexts. By delineating these boundaries, the present review fills a distinct gap by focusing on community and societal-level factors shaping loneliness and social isolation during typical social conditions.

Our findings reveal a striking imbalance in research focus, with only 3 included articles examining social isolation as a primary outcome compared with 10 addressing loneliness. The disparity likely reflects conceptual ambiguity regarding the distinctions between these constructs, the increasing prominence of loneliness within both scientific and public discourse, and potentially greater challenges in objectively measuring social isolation.^42^ With potentially different community and societal determinants and intervention pathways, the imbalance can have implications for practice, because loneliness and social isolation, although related, represent distinct experiences.

Despite clear recommendations from leading researchers to investigate these phenomena in tandem,^7^ our review demonstrates that the concurrent examination of both outcomes remains rare in young adult populations. This represents a significant epidemiologic gap that limits understanding of how community and societal factors might differentially influence objective social disconnection vs subjective experiences of loneliness. Interestingly, this pattern mirrors trends observed in research with older adult populations, whereby loneliness investigations significantly outnumber those for social isolation,^15^ suggesting a broader methodological bias within the field rather than an age-specific research tendency.

The measurement approaches for loneliness and social isolation observed in our included studies reflect broader methodological patterns and challenges within this emerging field. Validated multi-item scales such as the UCLA-LS and DJG-LS predominate in the loneliness literature,^43^ with the UCLA-LS demonstrating robust psychometric properties across diverse age cohorts and cultural contexts.^44^^,^^45^ However, our review revealed that single-item measures (eg, “Do you ever feel lonely?”) were used frequently, particularly in large-scale population surveys. Although pragmatic for data collection efficiency, these approaches may be more prone to underestimation of effect because they explicitly ask respondents to acknowledge loneliness, potentially triggering social desirability bias and stigma-related response distortion that multi-item scales can help mitigate.^46^ These measurement inconsistencies represent a significant methodological challenge for advancing the field and highlight the need for standardized, psychometrically sound approaches that balance practical implementation with conceptual validity.

The methodological limitations are even more pronounced for social isolation measurement. Using single-item questions to assess social isolation is particularly problematic, given its multidimensional nature, incorporating both network size and interaction frequency.^47^ The heterogeneity in measurement approaches further limited our ability to conduct meta-analyses. These measurement inconsistencies represent a key challenge for advancing this field and highlight the need for greater standardization in future research.

Our narrative synthesis revealed important insights about community and societal factors associated with loneliness and social isolation in young adults. Although most included studies demonstrated significant associations with community-level factors, Matthews et al.^24^ suggested that neighborhood characteristics (eg, neighborhood safety, social cohesion) may have relatively weaker associations with loneliness compared with interpersonal factors in their study. This discrepancy likely reflects both methodological differences and developmental considerations. Matthews et al.^24^ used a twin design that allowed control for shared familial and genetic liability to social factors. Although genetic factors do not directly determine neighborhood characteristics, such designs help account for unmeasured confounding related to heritable traits and shared family environments that may influence vulnerability to loneliness. Their study also exclusively examined 18-year-old individuals, potentially capturing a developmental period when community influences operate differently, compared with other life stages. Unlike other included studies that sampled across the broader young adult age range (18-30 years), participants in the Matthews et al.^24^ study were likely at a distinct life stage (eg, still living at home, transitioning from school) when family and peer relationships may exert stronger influences than neighborhood factors. This suggests that community-level determinants may have age-graded effects even within the young adult period, with potentially stronger community influences emerging as young adults establish independent households and develop place attachments outside the family home.^48^ Future research using longitudinal designs should explicitly examine age as a potential moderator within the young adult age range to better understand these developmental nuances.

The relationships between factors at different ecological levels represent an important consideration for understanding loneliness and social isolation. Most included studies adjusted for individual and interpersonal factors, such as age, gender, education, and income. Many continued to observe significant associations between community-level exposures and loneliness after accounting for these variables. However, the distinction between confounders and mediators is not always clear in the context of community-level determinants. For example, socioeconomic status may partially mediate the relationship between neighborhood context and loneliness, and adjustment for such variables may attenuate associations. Because this review relied on effect estimates as reported, we prioritized adjusted models where available, but we acknowledge that overadjustment cannot be ruled out and may contribute to heterogeneity in findings. Beyond statistical control, the interaction between ecological levels remains underexplored in the young adult population. Cross-level processes, such as whether strong interpersonal relationships buffer against negative community influences or whether certain individual vulnerabilities amplify the effects of neighborhood characteristics, were rarely examined directly. This ecological complexity highlights the need for research designs that can move beyond covariate adjustment to explicitly test moderating and mediating pathways across levels of influence.

Our findings highlight the important influence of cultural and societal norms on experiences of loneliness in young adults, in particular social inclusion and belonging. This is consistent with broader research on acculturation and mental health, which indicates that marginalization, characterized by disconnection from both heritage and host cultures, is associated with particularly poor mental health outcomes.^49^ Our research found that ethnic minority status,^23^ perceived discrimination,^23^ and residence in multicultural communities without perceived similarity to neighbors^26^ were all significantly associated with increased loneliness among young adults. These findings suggest that cultural displacement and marginalization may contribute to social disconnection through multiple pathways, underscoring the importance of culturally responsive approaches to loneliness prevention.

The identified relationships between cultural factors and loneliness point to important structural and systemic issues rather than simply individual adaptation challenges. The finding that residence in multicultural communities was associated with increased loneliness^26^ may initially seem counterintuitive but likely reflects complex dynamics related to social segregation, discrimination experiences, or perceived cultural distance that can persist even in demographically diverse settings. Evidence suggests that residents may experience social alienation within ethnically dense neighborhoods where integration and inclusion are limited, and that diversity can interact with broader structural inequalities, including economic stressors that weaken social cohesion and neighborhood connectedness. For example, Anicich et al.^50^ found that diverse neighborhoods were more likely to have exclusionary policies in public places like tennis clubs and golf courses, perpetuating segregation in America, whereas Finlay et al.^51^ demonstrated qualitatively that diverse public spaces can simultaneously increase exposure to difference while reinforcing perceptions of social distance.

Importantly, area-level classifications of multiculturalism may also capture broader contextual characteristics beyond cultural composition alone. Multicultural neighborhoods are frequently located within highly urbanized environments characterized by higher population density, residential mobility, and increased housing and cost-of-living pressures, which have been independently linked to reduced perceived neighborhood cohesion and lower community trust.^52^ Neighborhood change and population turnover may further limit opportunities for sustained community engagement and relationship formation in some populations.^53^ Structural pressures including financial hardship and differential access to community resources are also known to influence community engagement and neighborhood participation,^54^ both of which are strongly associated with loneliness outcomes. Collectively, these findings suggest that residual confounding by broader urban and structural conditions remains plausible, and that the observed associations should not be interpreted as reflecting cultural diversity per se but rather the complex social and structural environments in which diversity is situated. Furthermore, these findings highlight how diversity without inclusive social infrastructure can exacerbate rather than mitigate loneliness, suggesting that interventions must address not only individual cross-cultural competencies but also structural barriers to meaningful social integration.

The interplay between ecological levels was also evident in studies examining cultural and societal norms, where factors like individualism,^25^ and idealized masculinity^29^ demonstrated significant associations with loneliness. These cultural factors operate through complex pathways involving both societal expectations and individual internalization of these norms. For example, Goh et al.^25^ found that individualism was positively associated with loneliness, but this relationship was moderated by gender, with the association being stronger for young men than young women. This suggests that cultural orientation interacts with gender-based socialization patterns, potentially creating different vulnerability profiles. Similarly, Rovito et al.^29^ demonstrated that adherence to idealized masculine norms was positively associated with loneliness in young men, reflecting how societal gender expectations can constrain emotional expression and help-seeking behaviors, ultimately influencing personal experiences of connection. These findings align with theoretical perspectives on ecological systems that emphasize reciprocal interactions across levels rather than simple hierarchical influences.^55^

Comparative analysis of research outputs reveals less scholarly attention directed toward young adults relative to older populations. A scoping review from 2023 found 33 observational studies investigating community or societal factors in adults aged 60 years or older,^15^ compared with only 13 included in the present review, despite using comparable search methods and covering similar time periods. This research disparity likely stems from 2 distinct but potentially complementary explanations. First, there has been a historical assumption that older adults constitute the demographic most vulnerable to loneliness and social isolation,^11^ which has directed both research focus and funding priorities. Second, recent epidemiologic evidence suggests an actual demographic shift, with loneliness prevalence among young adults increasing over the past decade,^56^ creating an emerging public health concern to which research priorities have not yet fully adjusted to address. Regardless of which explanation predominates, young adulthood represents a critical developmental period characterized by transitions in education, employment, residence, and relationship formation. The limited research attention to date represents a significant missed opportunity to understand and address these issues during a formative life stage when intervention might prevent chronic patterns of disconnection.

### Limitations

Several methodological limitations should be considered when interpreting our findings. Our exclusive focus on quantitative, observational, and peer-reviewed literature, while methodologically appropriate for systematic review, potentially omits valuable insights from intervention studies, qualitative research, and gray literature. Policy documents and reports from not-for-profit organizations often contain relevant data on community-level determinants that may not reach academic publication; however, our methodological approach was justified by our primary aim to systematically assess the effects of community and societal determinants, which required standardized statistical reporting available predominantly in peer-reviewed quantitative research. Our deliberate exclusion of qualitative data was necessary to maintain methodological consistency, although it limited our ability to capture rich contextual factors. Future mixed-methods reviews could complement our findings by incorporating qualitative perspectives on mechanisms linking community factors to loneliness and social isolation.

The relatively small number of included articles (*n* = 13) reflects the emerging nature of this field. Our decision to include a broad range of community and societal factors, rather than restricting the review to predefined exposure categories, was conceptually aligned with the exploratory aim of the review, but may have affected search sensitivity. The diversity of terminology used to describe community and societal factors across disciplines required interpretive judgment during title and abstract screening to ensure relevant studies were not overlooked.

In addition, pragmatic decisions were required regarding age eligibility. Several population-based surveys report broader youth or early adulthood age bands that extend slightly beyond the target range of 18-30 years. Where substantial overlap with young adulthood was evident, such studies were retained to avoid unnecessary exclusion of otherwise relevant evidence. Although our inclusion criteria prioritized samples centered on young adulthood, residual age heterogeneity may have contributed to variability in findings. Future research would benefit from more precise age stratification within the young adult period to better capture potential developmental differences.

Although the majority of included articles were published in the past decade, several relied on data collected earlier, reflecting the secondary use of large population-based surveys. As a result, some community- and societal-level measures, including neighborhood cohesion, cultural norms, and objective indicators of social isolation, were operationalized using instruments developed in earlier periods. Although many of these tools remain widely used and psychometrically robust, they may not fully capture modern forms of social connection. This is particularly relevant in the context of digital communication, with present cohorts possessing greater digital maturity, changing the way they may connect with their peers.^57^ Additionally, contemporary life-course research shows that key social and biological transitions to adulthood, which may include labor market entry, education completion, and family formation, have shifted substantially over time, extending the social period of “young adulthood” and reshaping the contexts in which social connection and community participation occur.^58^ These considerations highlight the importance of interpreting findings within their historical and cohort contexts and underscore the need for longitudinal and cohort-comparative research using contemporary measurement approaches.

### Future research directions

Our findings highlight several critical directions for future research on community and societal determinants of loneliness and social isolation in young adults. Further longitudinal analysis would enable researchers to determine directionality and assess the temporality of the identified relationships, particularly for factors where reverse causality is highly plausible. Furthermore, the heterogeneity of the included articles represents a significant limitation to our ability to compare and contrast the findings. Methodological harmonization across studies, particularly in measurement approaches for loneliness and social isolation, would facilitate more robust meta-analytical synthesis and enable stronger comparative analysis across different contexts. Finally, there is a significant research gap in the assessment of these factors in relation to loneliness and social isolation concurrently, with only 1 included study taking this approach^32^; thus, so future studies should consider investigating these outcomes in tandem.

## Conclusion

Our study contributes to the theoretical understanding of loneliness and social isolation by applying a social ecological lens to an understudied age group, highlighting the complex interplay between individual experiences and broader structural contexts. The use of the social-ecological model to conceptualize the research approach to the prevention and reduction of loneliness and social isolation in young adults is supported by the available evidence; our findings point toward the multidetermined nature of these experiences. The use of the social-ecological model to explore community and societal level factors marks a departure from previous research, which has primarily focused on individual and interpersonal factors or on older adult populations. With influences identified across neighborhood, community, cultural, and structural domains, this framework appears well suited to capturing the complexity of factors that shape social connection and identifying potential intervention points. Our findings provide preliminary evidence that community-level factors spanning multiple domains, including neighborhood characteristics, community participation, social and cultural norms, and structural and systemic factors, are associated with experiences of loneliness in young adults, though evidence regarding social isolation remains limited. These findings point toward potential policy levers and community-based approaches that could complement individual-focused interventions. However, given the predominantly cross-sectional nature of available evidence, more research is required to ascertain the longitudinal impact and directionality of these community and societal determinants.

## Supplementary material

Supplementary material is available at *Epidemiologic Reviews* online.

## Supplementary Material

Web_Material_mxag007

## Data Availability

The authors confirm that all data generated or analyzed during this study are included in this published article.
